# Bacterial Adaptation during Chronic Respiratory Infections

**DOI:** 10.3390/pathogens4010066

**Published:** 2015-03-02

**Authors:** Louise Cullen, Siobhán McClean

**Affiliations:** Centre of Microbial Host Interactions, Institute of Technology Tallaght, Dublin 24, Ireland; E-Mail: Louise.cullen@postgrad.ittdublin.ie

**Keywords:** respiratory pathogens, bacterial adaptation, chronic infection, evolution, diversification, cystic fibrosis, chronic obstructive pulmonary disease (COPD)

## Abstract

Chronic lung infections are associated with increased morbidity and mortality for individuals with underlying respiratory conditions such as cystic fibrosis (CF) and chronic obstructive pulmonary disease (COPD). The process of chronic colonisation allows pathogens to adapt over time to cope with changing selection pressures, co-infecting species and antimicrobial therapies. These adaptations can occur due to environmental pressures in the lung such as inflammatory responses, hypoxia, nutrient deficiency, osmolarity, low pH and antibiotic therapies. Phenotypic adaptations in bacterial pathogens from acute to chronic infection include, but are not limited to, antibiotic resistance, exopolysaccharide production (mucoidy), loss in motility, formation of small colony variants, increased mutation rate, quorum sensing and altered production of virulence factors associated with chronic infection. The evolution of *Pseudomonas aeruginosa* during chronic lung infection has been widely studied. More recently, the adaptations that other chronically colonising respiratory pathogens, including *Staphylococcus aureus*, *Burkholderia cepacia* complex and *Haemophilus influenzae* undergo during chronic infection have also been investigated. This review aims to examine the adaptations utilised by different bacterial pathogens to aid in their evolution from acute to chronic pathogens of the immunocompromised lung including CF and COPD.

## 1. Introduction

The respiratory tract is the first point of contact in the lungs for pollutants and microorganisms, with, on average, 10,000 L of air inhaled per person per day. In healthy individuals, infections are rare despite bacteria being inhaled frequently, due to sophisticated host defence mechanisms at the lung mucosa. The combination of cilia and the mucous gel film on the epithelial surface allows for entrapment and clearance of particles and bacteria in healthy lungs. Antimicrobial peptides produced by epithelial cells and local immune responses such as secretory IgA and resident airway macrophages all contribute to the prevention of bacterial colonisation [1]. The healthy respiratory system has its own micro-flora, including *Staphylococcus epidermidis* and *Cornyebacteria*, *S. aureus*, non-haemolytic and alpha-haemolytic *Streptococci* and *Neisseria* species with occasional carriage of *Streptococcus pneumoniae* and *H. influenzae* in the upper airways [2]. The lower airway micro-flora, previously considered sterile, is now known to be colonised with a diverse range of genera including *Pseudomonas*, *Prevotella*, *Streptococcus*, *Fusobacteria*, *Veillonella*, *Haemophilus* and *Acinetobacter* species [3]. There are varying reports of the microbial communities in healthy lungs, and research into the respiratory microbiome in both healthy and disordered states is ongoing [4]. Understandably, the resident microbiome will depend on geography, climate and other environmental conditions [5].

Both cystic fibrosis (CF) and chronic obstructive pulmonary disease (COPD) are characterised by airway inflammation, altered mucus production and diminished mucocillary clearance. COPD and CF can cause bronchiectasis and both of these disorders are characterised by repeated cycles of inflammation, tissue damage and chronic bacterial infections contributing to a rapid decline in pulmonary function [6]. In a comparison of the COPD and healthy lung microbiome, the COPD state was found to differ in the levels of certain genera including *Afipia*, *Brevundimonas*, *Curvibacter*, *Moraxella*, *Neisseria*, *Corynebacterium*, *Undibacterium*, *Capnocytophaga* and *Leptolyngbia* [3]. This is in agreement with a comparison of the microbiomes in COPD and asthmatic patients with healthy controls, highlighting a diverse population of bacteria in the healthy controls. The composition of the microbiome of the healthy controls was considerably different from the diseased states [7]. The absence of functional cystic fibrosis transmembrane regulator (CFTR), a chloride ion channel, in CF patients results in airway surface liquid depletion [8]. CF sputum has a lower viscosity in comparison to asthma or bronchitis sputa but is highly tenacious leading to a reduction in cough clearance of infected phlegm, and subsequently inducing an inflammatory state in the airways [9]. A significant reduction in diversity of bacteria is also a hallmark of the CF microbiome. Older patients exhibit poorer lung function with more uneven and less diverse, more specialised communities, compared to younger healthy patients [10].

Alterations in the CF lung surface provide an environment which is exploited by CF pathogens. *P. aeruginosa* is the most prevalent pathogen that colonises people with CF. Another pathogen that chronically colonises the CF lung of predominantly adult patients is *Burkholderia cepacia* complex (Bcc), a group of 18 phenotypically similar, genetically distinct Gram negative bacterial species. The two most clinically relevant species are *B. cenocepacia* and *B. multivorans*. Other pathogens associated with CF lung infection include, *Haemophilus influenzae*, *Stenotrophomonas maltophilia*, *Achromobacter* *xyloxidans*, genus *Pandoraea* and Gram positive species including *S. aureus*, and Streptococci as recently reviewed [11]. These lung pathogens chronically colonise the CF lung contributing to gradual but unrelenting decline in pulmonary function and tissue damage [12]. COPD is characterised by restricted expiratory airflow from numerous anatomical lesions, including loss of lung elasticity, fibrosis and narrowing of the small airways. Inflammation, oedema, and secretions also contribute variably to airflow limitation. Smoking is a significant factor in COPD, inducing inflammatory responses, including oxidants and proteases, which play a major role in lung damage. In addition, cigarette smoke modifies lung repair responses in several ways [6]. Non-typeable *H. influenzae* is the most common coloniser of the COPD lung with *Moraxella catarrhalis* and *P. aeruginosa* being identified to lesser extents. *H. influenzae* is associated with persistent chronic infections, in contrast to *M. catarrhalis*, which is generally cleared within one month of acquisition [13]. *P. aeruginosa*, which chronically colonises the CF lung, is not frequently associated with persistence in COPD patients [14].

## 2. Bacterial Adaptation

**Figure 1:**
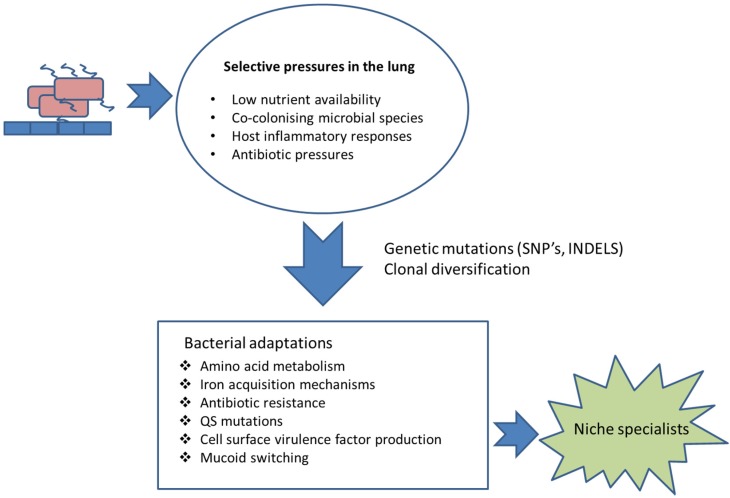
Selection and adaptation. Examples of selective pressures to which chronically colonising respiratory pathogens are exposed and the adaptations that they undergo, in order to enhance chances of survival.

Virulence factors or determinants are often non-essential to the pathogen but, if lost, they inevitably result in attenuated virulence [15]. Bacterial virulence factors are commonly in direct contact with the host or are involved in concealing the pathogen from the host immune system [16]. For example, outer membrane proteins (OMPs) are essential in adhesion, colonisation, intra-cellular invasion, antimicrobial resistance and intracellular communication processes [17]. The proteins can be considerably immunogenic [18]. In addition, the polysaccharide cell wall and capsule have anti-phagocytic properties required for immune evasion. Microbial secretory proteins are involved both in host microbe interactions and in tissue damage. The cell wall and outer membrane moieties such as lipopolysaccharide (LPS) are involved in recognition and host inflammation [16]. It is inevitable that in order for microbial pathogens and populations to chronically colonise a host, they need to adapt to the host environment and evolve over time within the host (Figure 1). The two key drivers for this are the enhanced survival and proliferation of the adapted pathogen to the host environment together with avoiding detection and destruction by the host immune system. Many, but not all, pathogens persist in the lung due to a down regulation of virulence factors and pathogen associated molecular patterns (PAMPS) to avoid detection by the host (Table 1). This review will focus on adaption of pathogens to the host lung during chronic colonisation. The majority of the research has focused on CF-associated pathogens; however, adaptations of bacteria associated with COPD have also been included.

**Table 1:** Bacterial adaptation during chronic infection: examples of adaptations and the general outcome during chronic respiratory infections.

Bacterial Component	Pathogen	Adaptation	Effect of Adaptation	Disease	References
Genome	*P. aeruginosa & S. aureus*	Mutations (single nucleotide polymorphisms, insertions, deletions)	Non-synonymous mutation effecting gene product	CF	[19,20]
Outer membrane proteins	*B. cenocepacia*	Upregulation of siderophore interacting protein, pyochelin receptor FptA, and TonB receptor	Iron chelating	CF	[21]
	*P. aeruginosa*	Phu mutations	Increased transcription and switch towards haemoglobin utilisation for iron	CF	[22]
	*H. influenzae*	Alteration of outer leaflet	Immune evasion	COPD	[23]
Lipopolysaccharide	*P. aeruginosa*	Loss of O-antigen	Immune evasion	CF	[24]
	*B. cenocepacia*	Reduced expression of enzymes involved in O-antigen and lipid a synthesis	Modifications in LPS and LPS expression	CF	[25]
Exopolysaccharide	*P. aeruginosa*	Non-mucoid to mucoid switch	Reduced virulence factor expression, poorer lung function in patients	CF	[26]
	*B. multivorans*	Mucoid to non-mucoid switch	Reduced *in vivo* virulence, reduction in virulence factor expression	CF	[27]
	*B. cenocepacia*	Mucoid to non-mucoid switch	Poorer outcome in patients	CF	[28]
Motility	*P. aeruginosa*	Loss in flagella associated motility	Phagocytosis evasion	CF	[29]
	*P. aeruginosa*	Loss of swimming, swarming and twitching	Loss of motility	COPD	[30]
	*B. cenocepacia*	Upregulation of *flhB*, *flhF*, *fliG*, *fliK*, *fliJ*, *fliN*, *flgB*, *flgD*, *flgE*, *flgF*, *flgG*, *flgH*, *flgI*	Increase flagellin assembly	CF	[31]
Colony morphotypes	*S. aureus*	Small colony variants	Antibiotic resistance, intracellular survival, reduction in α-toxin expression	CF	[32]
	*P. aeruginosa*	Rugose small colony variants	Increased auto-aggregative properties, increased biofilm formation	CF	[33]
	*B. cenocepacia*	Shiny colony variant	Reduced virulence and biofilm formation	CF	[34]
Quorum sensing	*P. aeruginosa*	*lasR* and r*hlR* mutation	Reduced production of QS associated virulence factors, increased resistance to β lactams, growth advantage with low levels of amino acids	CF	[35]
	*S. aureus*	*agr* mutation	Attenuated virulence, non-haemolytic	CF	[36]

### 2.1. Bacterial Genomic Evolution in the Host

The microbial genome evolves by a combination of point mutations, gene conversions and rearrangements, insertion of foreign DNA and deletions, enabling bacteria to adapt to the host environment. In addition, within an individual host, a clonal isolate can diverge forming separate, evolutionarily diverse species. Furthermore, the genetic information can be shared between pathogens via a horizontal gene transfer mechanism, further facilitating the adaptation of these opportunistic bacteria to their environments [37].

Investigations of the genomes of sequential isolates indicate that both *P. aeruginosa* and *S. aureus* are associated with reduced virulence over time of colonisation. In a shotgun whole genome sequencing study of *P. aeruginosa* sequential isolates from a CF patient over the course of eight years, it was clear that the isolates evolved within the host by a process of natural selection to reduce expression of virulence factors. There was a higher ratio of non-synonymous to synonymous mutations associated with an alteration in biological function. Mutations in genes regulating O-antigen biosynthesis, type III secretion, twitching motility, exotoxin A, multidrug efflux, osmotic balance, phenazine biosynthesis, quorum sensing (QS) and iron acquisition were all evident in the eight year isolate relative to the early isolate. The later isolate also expressed a mutation in *mutS*, a DNA mismatch repair gene, associated with increased mutation rates, which is potentially beneficial to the pathogen during long term colonisation [19].

In an attempt to determine the genetic within-host diversification *S. aureus* undergoes in the CF lung, a set of three sequential isolates, which were deemed identical based on sequence type, were examined over a period of 23 months. During the course of infection, 23 point mutations and 15 indels were identified; with 95.7% being non-synonymous mutations, consistent with niche adaptation of pathogens in CF. Mutations in *fusA*, associated with fusidic acid resistance was noted in the later isolate. In addition to this, the gene encoding the branched chain amino acid binding pocket of the global regulator CodY, normally involved in repressing cell virulence, contained a non-synonymous point mutation in this later isolate [20]. This later isolate had an increased ability to grow in threonine deficient media in comparison to the early and middle isolates, which has previously been associated with CodY mutants and is likely to be beneficial *in vivo* [38]. Multiple independent indel polymorphisms in all three isolates affecting SigB activity, a regulator of stress response, were also observed [20]. The parallel adaptation of mutations that influence virulence factor expression indicates a strong selective pressure on these loci contributing to a beneficial phenotype *in vivo*. In addition, a variation in the accessory genome in the later isolates was observed. Two distinct phage insertions were present in the initial isolate which encode secreted virulence factors, enterotoxin A and staphylokinase. The middle isolate and late isolates each lost one of these factors, illustrating a dynamic relationship between both the core and accessory genomes of *S. aureus* in suppressing virulence during chronic infection [20].

### 2.2. Diversification

While unique strains can be acquired during the early stages of infection, chronic colonisation is generally due to persistence of bacterial strains of the same lineage. The process of adaptation most likely occurs, one cell at a time, giving rise to sub-populations within the host. If these subpopulations can co-exist, then diversification of the population is inevitable. It is widely accepted that there is considerable heterogeneity and diversity across the *P. aeruginosa* population within the lungs of an individual with CF. This can be driven by a range of pressures including antibiotic treatment. Fothergill *et al.* [39] have shown significant fluctuations in morphotypes, virulence factors and antibiotic susceptibility within the *P. aeruginosa* populations during exacerbations and antibiotic treatment. In addition, genotypic variability arose within the *P. aeruginosa* population of each individual’s lung, predominantly due to phage activity. Variability in genotypes had also been reported by Smith *et al.* [19] in their earlier study of CF *P. aeruginosa* isolates over eight years, with as many as five genotypes (all from the same lineage), being identified in one sample from a three-year old patient. The extent of this diversification within a patient is highlighted by recent work by Workentine *et al.* [40] in their study of 169 isolates from a single patient. All isolates belonged to a single clonal group by pulse-field gel electrophoresis (with only two or three band differences), yet a high degree of phenotypic variability was observed even within isolates of the same colony morphotypes. A study of COPD colonised patients demonstrated that diversification of *P. aeruginosa* is not unique to CF patients and that different coexisted with different antibiotic susceptibilities within the individual patients for the entire period of the study [30]. Another study of COPD patients over 10 years showed that a minority of patients were chronically colonised with the same *P. aeruginosa* clone, but among these clonal diversification was evident [41].

Diversification is not exclusive to *P. aeruginosa* infection and has also been observed in Bcc colonised patients. A series of sequential clonal isolates recovered from a *B. multivorans* colonised patient colonised for 13 years showed diverse morphologies across the population [27]. *B. dolosa*, a relatively rare but considerably virulent member of Bcc showed distinct morphotypes within the same sputum sample [42]. Clonal isolates of *B. cenocepacia* isolated over 3.5 years of colonisation have also been shown to have diverged over time [21,25].

It has been recently demonstrated that adaptive mutations rarely fix within a patient’s population and that co-existence of diverse patients can exist for many years, adapting under the pressure of natural selection [43]. In an in-depth study of five CF patients chronically colonised with *B. dolosa*, Lieberman *et al.* showed that diverging populations coexisted for at least five years and that the diversification was driven by multiple adaptive mutations in the same genes evolving in parallel within an individual [43]. Diversification offers an insurance that facilitates persistence of the community as a whole during changes in environmental conditions [44].

### 2.3. Hypermutability

Hypermutable microbes have an increased spontaneous mutation rate, as a result of defects in the DNA repair system or proof reading systems. These mutators play a major role in the evolution of the pathogen over time [45]. Antibiotics select for these variants, as these undergo more genetic mutations and are better able to adapt and survive under the antimicrobial pressures *in vivo* [46]. It has also been shown that the host environment can also select for mutator microbes, within *P. aeruginosa*, *S. aureus* and *H. influenza* populations [45]. The hypermutable strains of *H. influenzae* are more commonly isolated from the CF host than non-CF patients, which is most likely due to the high levels of antimicrobial therapy administered to these individuals. Nevertheless these mutator strains confer a long term persistence advantage [12,47]. Interestingly these mutators are rarely isolated in acute infections [45].

Hypermutable *P. aeruginosa* strains become more frequent in later stages or chronic infection. Mutations in *mutS*, *mutL* and *uvrD* genes encoding proofreading proteins which normally correct errors during DNA replication give rise to these hypermutable strains [12]. It is important to note that the increase in hypermutable *P. aeruginosa* isolates later in CF patients suggest that genetic and phenotypic diversification plays an essential role in the adaptation of *P. aeruginosa* to the hostile and diverse CF lung environment and plays a role in survival *in vivo* by selecting for less virulent phenotypes [48].

Yang *et al.* carried out a comprehensive whole genome sequencing analysis of the transmissible *P. aeruginosa* DK2 lineage over a period of 35 years (200,000 bacterial generations) in CF patients *in vivo* [49]. From this extensive analysis of sequential isolates an initial period of niche adaptive evolution was evident. Over a 35 year period a total of only 180 SNP’s occurred. The later isolates may be dominated by negative selection which has lasted and remained within the lineage. The authors further state that the resulting success of this lineage and its ability to thrive and survive long term *in vivo*, and its ability to spread from patient to patient may be explained by the adapted evolutionary mechanisms employed by the pathogen [49,50].

## 3. Adaptations in Surface Molecules

### 3.1. Adaptations in Outer Membrane Proteins

The outer membrane proteins (OMPs) are key players for within-host adaptive strategies of these Gram negative bacteria. Many OMP’s are regulated by environmental factors, some of which enhance the adaptability of bacterial pathogens to the host environment and during the infection process. The majority of OMPs are composed of monomeric or trimeric barrels comprised of 8–22 anti-parallel β-strands [17]. Membrane proteins that are involved in iron uptake are necessary for survival and virulence. Bacteria possess the ability to adapt to the low levels of iron in the host lungs by production of siderophores or iron chelating compounds which cleaves the hosts iron and utilises it as a co-factor. These are generally upregulated during chronic infection *in vivo*, in iron sparse environments. Proteomic expression studies suggest that up regulation of these iron chelating compounds by *B. cenocepacia* increases over time in the lung [21]. Deletions of these proteins in *B. cenocepacia* showed reduced virulence *in vivo* implicating the role of these proteins in virulence [51,52]. In terms of evolutionary advantage, enhanced survival via iron acquisition must overcome the potential risk of increased host detection. Active outer membrane transporters such as TonB and TonB dependent proteins also have a siderophore uptake function in the iron limited host environment, for example FpvA in *P. aeruginosa* [17,53]. Promoter mutations in the *phu* system which encodes the OM receptor, PhuR, the periplasmic soluble receptor, PhuT, and the permease, PhuUV, were identified in sequential clinical *P. aeruginosa* isolates [22]. This allowed increased transcription of these genes and an adaptation towards haemoglobin utilisation within the host.

The cell capsule OMPs have been implicated in serum resistance in the host, providing the bacterium with a means to evade the complement mediated killing. Nakamura *et al.* hypothesised that *H. influenzae* adapts to inflammation encountered during infection of the lower respiratory tract in COPD patients by modulation of its outer leaflet through increased expression of *vacJ* and *yrb* genes in order to minimize recognition by bactericidal anti-oligosaccharide antibodies [23].

### 3.2. LPS

LPS is an important glycolipid PAMP embedded in the outer cell leaflet of Gram negative bacteria. It induces a variety of responses in the host upon binding to TLR4, TLR2 and CD14 including pyrogenicity, platelet aggregation and induction of cytokines [54,55,56]. LPS composition has also been implicated in serum resistance [57]. LPS has three components: the toxic, highly acylated Lipid A; the central core oligosaccharides containing unusual sugars and the O-antigen. LPS composition increases asymmetry in membrane architecture, and the subsequent cross linking of LPS with divalent cations decreases the permeability to hydrophilic agents which increase the level of innate antibiotic resistance to these agents [58].

During chronic CF infection *P. aeruginosa* LPS structure is altered, with the loss or diminished production of O-antigen being frequently reported in later chronic stages of the disease [24]. These adaptations have recently been reviewed by Hauser *et al.* (2011) [12]. The immuno-stimulatory properties associated with the O antigen confer a selective advantage to cells lacking this structure. The fact that *P. aeruginosa* blood isolates are not frequently reported (in contrast to Bcc) could be as a result of the O-antigen structure alteration during chronic infection [59]. Proteomic profiling using 2-D DIGE studies have shown that O-antigen biosynthesis is also reduced in later sequential *B. cenocepacia* isolates. Both the expression of ManB (a phosphomannomutase) and an NAD-dependent epimerase, involved in lipid A and O-antigen synthesis, respectively, were lower in the later isolate taken close to patient death relative to an early isolate [21]. Two forms of the outer membrane assembly factor YaeT which is involved in maintaining a homeostatic LPS ratio was also up-regulated in the later isolate. This suggests that like *P. aeruginosa*, *B. cenocepacia* isolates can reduce the production of the immuno-stimulatory O-antigen and lipid A composition of the LPS in the later stages of infection, which could potentially benefit the pathogen *in vivo* [21].

### 3.3. Exopolysaccharide (EPS)

A mucoid phenotype is one of the adaptations that some pathogens undergo during respiratory infection. Mucoid colony morphology in *P. aeruginosa* most commonly occurs by overproduction of EPS alginate, a polymer of d-manuronic acid and l-glucuronic acid [60]. During initial infection of the CF lung, *P. aeruginosa* isolates are typically non-mucoid while later isolates are more mucoid in morphology as a result of mutations in the *mucA* gene encoding an inner membrane associated anti-σ-factor [26]. *MucA* normally limits the expression of the *algD* operon, encoding the enzymes required for alginate production and numerous stress responsive virulence genes by sequestering the alternative anti-σ^22^-factor encoded by *algU* [61]. Mutated *mucA* leaves this anti-σ^22^ free to activate many genes including genes involved in alginate production. This conversion in gene expression is controlled by the *algU/algT* regulon [62]. Remarkably the activation of this regulon also causes synchronised down regulation of central metabolism, motility and virulence and a simultaneous up-regulation in genes affecting membrane permeability and antibiotic efflux [50], all of which would prove advantageous for the bacterium during chronic lung infection. The genetic mutations that bring this about occur as a result of cell envelope stress during unfavourable conditions such as osmotic shock, oxidative stress, magnesium starvation and antimicrobial agents in the inflammatory COPD and CF lung [50,63].

Clinical mucoid sequential clones of *P. aeruginosa* showed reduced virulence over time, in contrast to the non-mucoid clones which were more virulent *in vivo* and were associated with reduced clearance [64]. Late non-mucoid isolates of *P. aeruginosa* have also been obtained from the CF lung; these are suspected revertants of mucoid isolates indicating that the *in vivo* conditions have changed [65]. The mucoid phenotype switch in *P. aeruginosa* during chronic infection is associated with poor lung functions, increased anti-EPS antibody titres and poorer outcomes in CF patients [59]. In addition, Huse *et al.* (2013) demonstrated that non-alginate EPS by *P. aeruginosa* is just as important as alginate EPS during adaptation to the CF lung [66]. Operons undergoing parallel evolution *in vivo* enhanced the production of galactose-mannose rich EPS (Psl) in the first 40,000 generations [66,67]. The later chronic clinical isolates produced more Psl than their ancestor strains in 70% of cases *in vivo*. Psl is required for biofilm formation in mucoid isolates, furthermore, small colony variants overproducing Psl underwent positive selection during chronic colonisation [66].

Zlosnik *et al.* (2008) showed that mucoid phenotype switches also occurred in sequential isolates from 15 patients with Bcc infection [68], although in contrast to *P. aeruginosa*, the majority of Bcc isolates showed a mucoid to non-mucoid switch. The non-mucoid Bcc isolates were associated with a more rapid lung function decline than mucoid isolates [69], indicating that this mucoid switch may be associated with increased virulence. In a follow-up study, it was shown that non-mucoid *B. cenocepacia* isolates were associated with elevated expression of several putative virulence factors. Mutations over time occurred in the *manC* gene of Bcc [28], which is associated with both EPS and O-antigen synthesis [70]. Mutations were also observed in *wpbA* and *wpbW*, which are *manC* homologues in *P. aeruginosa* and are associated with reduced O-antigen synthesis in *P. aeruginosa* over time of colonisation. This suggests that the same forces driving adaptation may also be implicated in niche adaptation within the CF lung in different pathogens [70].

Another Bcc species, *B. multivorans*, also showed a mucoid to non-mucoid switch, but in contrast to *B. cenocepacia* and more comparable with *P. aeruginosa*, showed reduced virulence over time, with the later non-mucoid isolate being less virulent than the clonal mucoid isolate [27]. Reductions in swimming and swarming motility, type IV secretion, haemolysin-type protein secretion and EPS production over time were also observed [27]. In the examination of the structural properties of EPS from Bcc isolates, Herasimenka *et al.* (2007) found that cepacian was the most common EPS produced by a set of seven clinical isolates including three *B. cenocepacia*, three *B. multivorans* and one Bcc member of which taxonomic status had not yet been determined. Only one *B. multivorans* isolate produced psl, however, no definitive evidence to suggest the presence of cepacian EPS in sputum was noted [71]. It has been suggested however that the production of EPS by *B. cenocepacia* does confer a selective advantage *in vivo* by the inhibition of neutrophil chemotaxis and reactive oxygen species [72].

Capsular polysaccharide expression in chronic *S. aureus* infection was significantly reduced in later isolates relative to earlier isolates, which may be involved in this pathogen’s persistence *in vivo* [73]. Several chronic infection models of *S. aureus* indicated altered virulence factor expression over time of colonisation. Proteins associated with host protein interactions (Fbp1) were elevated while the virulence protein haemolysin was reduced indicating enhanced potential to colonise and avoid immune detection [73].

## 4. Antibiotic Resistance

Antibiotic resistance is a hallmark of chronically colonising pathogens generally and particularly in those associated with CF infections [74,75,76]. Bacteria acquire and utilise different antibiotic resistance mechanisms to protect themselves *in vivo* including; efflux pumps, porins and altered membrane permeability, enzymatic modification such as β-lactamases which have previously been reviewed [58,77,78]. These resistance mechanisms can be altered during the *in vivo* course of infection and by exposure to a range of antimicrobial agents, in addition to patient non-compliance [74]. Studies have reported the increased antibiotic resistance of many pulmonary pathogens to a variety of therapeutics over time *in vivo* [30]. Antibiotic resistance has been associated with modifications in LPS, biofilm formation and QS mutations in *P. aeruginosa* during chronic infection [12]. OMP’s and in particular multi drug efflux pumps can increase antimicrobial tolerance. These proteins confer resistance to many antibiotics after exposure *in vivo* which have previously been reviewed [79].

During antibiotic treatment of *P. aeruginosa* in CF patients, mutations arose in the genes encoding β-lactamases and efflux proteins which confer higher levels of antibiotic resistance. This can potentially be associated with decreased lung function in CF patients over time, but this is not always the case [12,80]. *P. aeruginosa* isolates from COPD patients also showed a general increased resistance to antibiotics over time. As has been discussed (Section 2.2) antibiotic susceptibility is diverse within single sputum isolates with many morphotypes evident, both sensitive and resistant, and with a wide range of variability among the sensitive isolates [40].

Bacteria from the Bcc are also highly antimicrobially resistant and resistance patterns differ depending on their environmental conditions. As with *P. aeruginosa*, highly resistant strains of Bcc are isolated from the CF lung more frequently than from non-CF patients. This is most likely as a direct consequence of *in vivo* adaptation of the microbe to the high levels of administered biocidal agents over time [81]. Early *B. cenocepacia* clonal isolates were more susceptible to cephalosporins, carbapenem, aminoglycosides, fluoroquinalones and trimethoprim-sulfamethoxazole than subsequent later isolates [82]. In contrast, a late *B. multivorans* isolate was more susceptible to β-lactam antibiotics, which is not frequently observed in clinical isolates exposed to antibiotics *in vivo* [27]. It was reported that this may be due to accumulated strain specific mutations and that it is unlikely to be clinically relevant.

Methicillin resistance *S. aureus* (MRSA) is also a causative agent in nosocomial acquired pneumonia, which has severe implications for individuals with underlying pulmonary disorders such as CF or COPD [83,84]. The small colony variant (SCV) phenotype of *S. aureus* which is a common phenotypic change during infection in CF patients has been implicated in antibiotic resistance, in particular to aminoglycoside antibiotics, as these SCVs lack an efficient electron transport system, which is required for the antibiotic to enter the cells [12,85]. In addition to this the ability of *S. aureus* SCV’s to survive within host cells reduces the efficacy of therapeutics against this pathogen [86].

### Antibiotic Stress-Effect on the Airway Microbiome

During antibiotic treatment of CF lung infections, it is essential to consider the effect of the biocidal therapy on the co-colonising microorganisms and indeed the lungs’ own microflora. Zemanick *et al.* [84] compared inflammatory markers and bacterial species in 21 patients with pulmonary exacerbations at early stage (0–3 days) and late stage (7–14 days) of antibiotic treatment. The relative abundance of *P. aeruginosa* and not anaerobic genera was associated with lower FEV_1_ values and higher levels of inflammation during antibiotic therapy. In addition, the higher abundance of *P. aeruginosa* was associated with lower microbial diversity, poorer lung function and increased inflammation, suggesting that the alterations in relative abundance balance of these core anaerobic species, such as *Prevotella* and *Vellionella*, may contribute to pulmonary disease [87]. Disruption of the pulmonary microbiome with high levels of biocidal therapies has also been suggested for patients with asthma and COPD [7,88]. Tunney *et al.* observed that targeted treatment of aerobes had minimal effect on the abundance and diversity of co-colonising anaerobes [89]. More recently, it has been reported that the relative abundance of *P. aeruginosa* increased in comparison to non-pseudomonal species during antibiotic therapy with anti-pseudomonal antibiotics [90].

## 5. Motility

A common switch from a highly motile phenotype to a non-flagellated, non-motile variant in *P. aeruginosa* was reported 20 years ago [91]. This loss in flagella was associated with mutations in flagellar genes including *rpoN*, *vfr*, *fleQ* and *fliC*. Although the adaptation to a non-motile phenotype during chronic infection was not associated with poorer outcomes, the inability of macrophages to phagocytose non-flagellated *P. aeruginosa* isolates suggested a potential reason for this phenotypic selection *in vivo* [29]. More recent studies analysing *P. aeruginosa* evolution in COPD patients over time of colonisation support this observed reduction in swimming, swarming and twitching motility in chronic infections [30]. Both swimming motility and swarming motility have been associated with biofilm formation [92]. Patankar *et al.* (2013) demonstrated that this loss in motility caused a reduced inflammasome activation and antibacterial IL-1β host response and could potentially explain how pathogens have evolved strategies to avoid host immune attack [93]. Transcriptional analysis of *B. multivorans* also showed a decrease in gene transcripts associated with motility and chemotaxis in a late clinical isolate [27]. Conversely numerous *B. cenocepacia* genes associated with flagella assembly and adhesion were more highly expressed in a later variant when compared to the earlier isolate [31]. In agreement with this a recent study of 551 Bcc isolates from chronic infections, showed that swimming motility was not lost by Bcc, in contrast to *P. aeruginosa* [94].

## 6. Morphology Variants

Many bacteria alter their morphology over time of colonisation. In particular, SCVs have a much slower growth rate, which results in smaller colony morphology when grown on routine agar [95]. These SCV’s are often selected for by prolonged antibiotic exposure [65,96], which is common during chronic lung infections. *S. aureus* SCV’s are associated with persistence in the airways. These slower to grow, antibiotic resistant, non-pigmented colonies also alter their virulence factor expression, for example, reduced expression of α-toxin in *S. aureus* SCV’s. The high expression of fibronectin binding proteins (FnBPs) in SCV *S. aureus* promotes their own uptake in host cells which proves beneficial [32]. Once located intracellularly these SCV’s avoid immune activation by reduced host tissue damage and bacterial protease secretions. They are better adapted for intracellular survival in higher numbers and better able to avoid and withstand antimicrobial activity elicited by host cells than their wild type counterparts. The dynamic ability of SCV’s to revert to their wild type phenotype also play a role in chronic infection and aid in the pathogens ability to chronically infect its host [32]. This persistence is potentially related to their greater resistance to host immune defences and antibiotics and their greater ability to adhere to respiratory epithelial cells [97].

In *P. aeruginosa*, SCVs can also exhibit hyperadherent and autoaggregative behaviour which can favour biofilm formation and persistence *in vivo* [98,99]. *B. multivorans* and *B. cepacia* also express the SCV phenotype which has led to fatal outcomes after bilateral lung transplantation in two patients out of three with CF [100]. The SCV morphotype of these Bcc isolates demonstrated an increased resistance to serum relative to the wild type. Bcc have also been shown to undergo shiny colony variant (SHV), with a switch from a matte colony appearance to SHV which was associated with persistence in mice [101]. This conversion has been associated with reduced virulence and biofilm formation in *B. cenocepacia* K562, with SHV producing significantly less lung histopathology than the rough K562 strain in a mouse agar bead model of chronic infection [102]. Other pleiotropic effects occur including reduction in motility, biofilm formation, extracellular matrices and siderophores noted [103].

Rugose small colony variants (RSCV) of *P. aeruginosa* CF isolates have been examined in a study by Starkey *et al.* (2009) using a combination of transcriptional profiling and Biolog phenotypic analysis. RSCV’s are small wrinkled colonies that have an increased auto-aggregative and biofilm formation capacity. RSCV’s selected for during persistent infection showed increased expression of the *pel* and *psl* polysaccharide gene clusters and decreased expression of flagellum and pilus genes [19,33].

## 7. Quorum Sensing

Prokaryotes generally behave as single cellular organisms in low population densities, however once they sense that the population density has reached a desired level in the formation of biofilms. This QS process allows cells to communicate with each other using small signalling molecules, autoinducers and enables the bacteria to alter the expression of different virulence genes essential for pathogenicity. Mutations in two QS systems LasR and RhlR, which result in loss of QS in clinical *P. aeruginosa* isolates, were more frequently identified later in CF infection. These QS mutants demonstrated a growth advantage with low levels of amino acids which is particularly relevant in a pulmonary environment and over time of chronic infection *in vivo* [35,104]. Since virulence factors are generally selected against during chronic infection *in vivo*, and as QS controls a variety of different virulence factor expression, QS mechanisms could be potentially detrimental to the bacterium during chronic infection [12]. Another advantage in down regulation of QS mechanisms could be attributed to *P. aeruginosa* QS mutants having increased β-lactamase activity *in vitro* [35]. In an attempt to examine the social behaviour of *P. aeruginosa* during chronic infection Jiricny *et al.*, (2014) showed a statistically significant reduction in the 3-oxo-C12-HSL and Pseudomonas Quinalone Signal (PQS) systems [105].

In contrast to *P. aeruginosa*, McKeon *et al.* showed that *B. cenocepacia* QS mutants were far less frequent during chronic infection. Only 1 in 22 patients harboured mutations in both QS Bcc systems, cepR and cciR, which would have an effect on function, the loss in QS function did not appear to confer a competitive advantage for *B. cenocepacia in vivo* [106]. Recent data showed that a triple knock-out mutant in J2315 of cepl, cciR and *Burkholderia* diffusible signal factor, had reduced biofilm forming capacities, was more susceptible to antibiotics, and was less virulent in *C. elegans* than the wild type strain [107], which may explain the lack of QS mutants in chronically colonised CF patients.

*S. aureus* utilise an accessory gene regulator (*agr*) QS system which is essential for pathogenesis, biofilm formation and adhesion. *S. aureus* also utilises a LuxS system in biofilm formation [108]. Interestingly *agr* mutants are common in chronic CF infection, these mutants are often non-haemolytic with attenuated virulence that cannot lyse blood cells [36].

## 8. Interactions with Co-Colonising Fungi

In pulmonary diseases including CF and COPD it is accepted that fungal infections including *Aspergillus* and yeast *Candida* species are frequently observed and are associated with varied outcomes in the patients infected [109,110]. The effect of co-colonising fungi on pathogenic bacteria cannot be overlooked. In a study of chronically infected CF patients *A. fumigatus* was identified in 45.7% of patients and significantly *C. albicans* was isolated from 75.5% of patients [111]. Interestingly an indirect correlation between the richness and diversity of both bacterial and fungal species in the lungs of CF patients and poorer clinical outcomes has been observed, highlighting the role of both commensal and opportunistic pathogens and fungi in pulmonary diseases [112]. The interactions of bacterial and fungal species are evident in the biofilm structures formed *in vitro* during co-colonisation with *P. aeruginosa* and *A. fumigatus*. *P. aeruginosa* alone formed loosely adherent biofilms in monoculture, however when co-cultured with already pre-established *A. fumigatus* mycelia, firmly adherent biofilm structures were observed which were comparable with *A. fumigatus* biofilm structure alone [113]. Mowat *et al.* subsequently showed diffusible factors produced by *P. aeruginosa* could inhibit and disrupt biofilm formation of *A. fumigatus* [114]. Furthermore, the extent of *P. aeruginosa* induced lung injury in BALB/c mice was shown to be reduced when preceded by airway colonisation by *C. albicans*, an effect that was reversed by treatment with the antifungal, capsofungin. This highlights *C. albicans* could modulate the virulence of *P. aeruginosa* in an *in vivo* model again highlighting the complex relationship between these species *in vivo* [115].

## 9. Methodologies Exploited in the Study of Adaptation

### 9.1. Microarrays in the Study of Microbial Adaptation and Pathogenesis

In the last few decades major advances have been made in the study of single genes to whole genome sequencing in pathogens. A number of genomic tools have been used to determine virulence factors involved in pathogenesis. With increasing numbers of bacterial genomes published, comparative genomics and expression analysis has come to the fore. In particular, microarrays have been powerful tools in the determination of alterations in gene expression during the course of clinical infection or comparison of virulence determinants in different lung pathogens. The use of cDNA microarrays allows the rapid elucidation of gene expression divergence among sequential clinical isolates during the course of infection. The analysis of genes involved in virulence can be performed on numerous pathogens simultaneously, which is advantageous when dealing with large numbers of sequential isolates. The evolution of bacterial genetics at the DNA level can also be studied using these robotic chip-based systems [116]. Microarrays and independent component analysis proved useful in determining that *P. aeruginosa* utilises multiple patient specific and parallel adaptations during chronic CF infection. The common parallel adaptations revealed that earlier isolates had higher levels of type three secretion systems and exoenzyme activities than later isolates, whereas the later isolates generally shared antimicrobial peptide resistance genes, alginate production genes and QS genes [117]. Other applications of this technology in elucidating certain adaptations that pulmonary pathogens undergo during chronic infection have already been discussed in this review.

The limitation associated with this technology is that robotics and array fabrication can be variable and data analysis complex. Careful designing of an experiment, improved system components and quality control steps at each step of the process can eliminate these variables. Importantly microarray analysis on DNA and mRNA transcripts levels does not fully depict the protein expression levels within a cell and the gene function in microbes, with protein translations and modification all impacting on the final functional molecules in the cells. Nevertheless this technology has provided great insight into the ability of microbes to diversify during chronic lung infections [118].

### 9.2. Proteomics in the Study of Microbial Adaptation and Pathogenesis

The utilisation of proteomic approaches in bacterial evolution has been discussed throughout this review. Madeira *et al.* utilised a 2D Difference gel electrophoresis (2D DIGE) to determine evolution strategies employed by three sequential CF *B. cenocepacia* isolates from a total of 11 isolates. These three isolates included the initial isolate and two later isolates (one following an exacerbation and one IST4134 retrieved 3.5 years later immediately prior to patient death). The proteomic analysis correlated well with alterations in phenotype. The two later isolates were more capable of invading and disrupting 16HBE14o- and CFBE41o-cells *in vitro* and were also better able to cope in iron limited conditions which collectively shows a beneficial adaptive strategy employed by the pathogen to survive over time in the CF lung. A metabolic reprogramming was also speculated [21]. In an earlier study by Chung and Speert (2006) a combination of 2D gel and MALDI-ToF mass spectrometry were utilised to determine the proteins necessary for *B. cenocepacia* survival in a murine host, they noted that alkaline hydroperoxide reductase subunit C (ahpC) protein spot was lost in the persistent isolate. AhpC expression has been associated with resistance to oxidative stress. An increase in flagellar genes was also observed, which may be associated with bacterial persistence [119]. Gel based systems provide a relatively straight forward method to study proteomics; however with the development of MS to complement these approaches gel based proteomics are getting less scope recently. Limitations associated with gel based proteomics include poor representation of low abundant proteins, difficulty to resolve and separate highly acidic/basic/hydrophobic and large proteins. In addition to this the potential for multiple proteins to be present in a single spot can result in inaccurate comparisons [120]. LC/MS proteomic platforms avoid some of these issues and have been successful in examining non-protein QS signalling molecules, for example [105].

Limitations to this type of analysis include; total accurate quantification of a particular protein is only possible when the peptides are exclusively derived from a particular protein. In addition to this quantification of post translational modifications is still developing [121]. Nevertheless this platform has proved extremely valuable in determining pathogen adaptation during chronic lung infection.

## 10. Conclusions

Chronic lung infections are associated with increased morbidity and mortality for individuals with underlying pulmonary disorders, particularly for CF and COPD patients. The pathogenesis and adaptation mechanisms utilised by Gram positive and Gram negative bacteria during chronic lung infection involves numerous complex and diverse virulence factors. These determinants include surface components that are in direct contact with the host such as adhesins and outer-membrane proteins and pyrogenic LPS involved in the initial colonisation step. Antibiotic resistance, biofilm formation, EPS production, hypermutability and colony variants, which are frequently reported during chronic infection can be altered through the clinical course of infection to assist in the survival and persistence of these pathogenic pulmonary pathogens. It is becoming apparent that while *P. aeruginosa* and other species, such as *B. multivorans* reduce their virulence over time, *B. cenocepacia*, contrary to expectations, appears to increase its virulence (for example, non-mucoid phenotype, flagella). The expression and repression of these virulence factors during chronic infection involves an intricate regulation system unique to a bacterial species, isolate or cell that ultimately provides the pathogen with a competitive edge *in vivo*. These systems include QS systems and mutations in genes responsible for various virulence factor regulations, for example MucA. The frequency of mutations in *mucA* and QS in *P. aeruginosa* would suggest that loss of function of these virulence regulatory genes is advantageous *in vivo* and selective pressures within the host select for these mutants.

The evolution of bacteria is extremely complex and cannot be explained by a single evolutionary trajectory. It is more plausible that numerous simultaneous and parallel mutations select for pathogenic communities that are better suited to their niche. The host lung environment, co-infecting microbes and cell specific factors all contribute to the diversity of these pathogens, which makes extrapolation and comparison of these opportunists difficult. Nevertheless these pathogens have one common goal, which is to survive within the host lungs by evasion of host defences and antimicrobial therapies. Further studies in this area using a combination of molecular, proteomic and phenotypic studies will contribute to the knowledge of bacterial pathogenesis and evolution and provide potential therapeutic targets in eradicating these frequently antibiotic resistant pathogens.
